# Differences in the lipid metabolism profile and clinical characteristics between eosinophilic and non-eosinophilic acute exacerbation of chronic obstructive pulmonary disease

**DOI:** 10.3389/fmolb.2023.1204985

**Published:** 2023-07-12

**Authors:** Yating Wang, Chun Chang, Sifan Tian, Juan Wang, Xiaoyan Gai, Qiqiang Zhou, Yahong Chen, Xu Gao, Yongchang Sun, Ying Liang

**Affiliations:** ^1^ Department of Respiratory and Critical Care Medicine, Peking University Third Hospital, Beijing, China; ^2^ Research Center for Chronic Airway Diseases, Peking University Health Science Center, Beijing, China; ^3^ Department of Occupational and Environmental Health Sciences, School of Public Health, Peking University, Beijing, China

**Keywords:** chronic obstructive pulmonary disease, acute exacerbation, eosinophils, lipidomics, LC-ESI-MS

## Abstract

**Objective:** In this study, we aimed to investigate the differences in serum lipid metabolite profiles and their relationship with clinical characteristics between patients with eosinophilic and non-eosinophilic AECOPD.

**Methods:** A total of 71 AECOPD patients were enrolled. Eosinophilic AECOPD was defined as blood EOS% ≥ 2% (*n* = 23), while non-eosinophilic AECOPD, as blood EOS< 2% (*n* = 48). Clinical data were collected, and serum lipid metabolism profiles were detected by liquid chromatography–mass spectrometry (LC-MS). The XCMS software package was used to pre-process the raw data, and then, lipid metabolite identification was achieved through a spectral match using LipidBlast library. Differences in lipid profiles and clinical features between eosinophilic and non-eosinophilic groups were analyzed by generalized linear regression. The least absolute shrinkage and selection operator (LASSO) was applied to screen the most characteristic lipid markers for the eosinophilic phenotype.

**Results:** Eosinophilic AECOPD patients had less hypercapnic respiratory failures, less ICU admissions, a shorter length of stay in the hospital, and a lower fibrinogen level. In the lipid metabolism profiles, 32 significantly different lipid metabolites were screened through a *t*-test adjusted by using FDR (FDR-adjusted *p* < 0.05 and VIP> 1). Nine differential lipid metabolites were found to be associated with the three clinical features, namely, hypercapnia respiratory failure, ICU admission, and fibrinogen in further integration analysis. The species of triacylglycerol (TAG), phosphatidylcholine (PC), lysophosphatidylcholine (LPC), and diacylglyceryl trimethylhomoserine (DGTS) were high in these eosinophilic AECOPD. The LASSO was applied, and three lipid metabolites were retained, namely, LPC (16:0), TAG (17:0/17:2/17:2), and LPC (20:2). The logistic regression model was fitted using these three markers, and the area under the ROC curve of the model was 0.834 (95% CI: 0.740–0.929).

**Conclusion:** Patients with eosinophilic AECOPD had a unique lipid metabolism status. Species of TAGs and LPCs were significantly increased in this phenotype and were associated with better clinical outcomes.

## 1 Introduction

Chronic obstructive pulmonary disease (COPD) is a heterogeneous disease characterized by persistent respiratory symptoms and airflow limitations (Global Initiative for Chronic Obstructive Lung Disease, 2022). It is estimated that there are nearly 100 million patients with COPD in mainland China, with a prevalence of 13.7% among adults aged ≥ 40 years (Wang et al., 2018). The prevalence of COPD will continue to rise, adding a heavy economic burden to individuals and society (Global Initiative for Chronic Obstructive Lung Disease, 2022). Additionally, acute exacerbations of COPD (AECOPD) have a significant impact on patients’ health (Wilkinson et al., 2004), contributing to hospitalization and readmission, disease progression, and increased risk of death.

Eosinophilic COPD is a phenotype of airway inflammation characterized by the presence of eosinophilic inflammation in the airways, manifested by elevated peripheral blood and/or sputum eosinophil counts (Barnes, 2019). Some patients with COPD during exacerbation also have a peripheral blood eosinophilia ≥ 2% or sputum eosinophilia ≥ 3%, who are classified as eosinophilic AECOPD phenotypes (Singh et al., 2014; Pascoe et al., 2015; Dai et al., 2020). These patients have specific characteristics in terms of clinical characteristics, laboratory test results, treatment, and prognosis. For instance, the risk of in-hospital mortality is lower among eosinophilic AECOPD patients, and the hospital stay is shorter (You and Shi, 2021), with better response to systemic glucocorticoid treatment (Bafadhel et al., 2014). However, the pathogenesis of the eosinophilic phenotype of COPD exacerbation is still not clear. Novel biomarkers can help clinicians recognize this phenotype and develop potential new therapeutic targets.

Lipids are important cellular components. They participate in the formation of cell membranes and are important energy storage substances, components of various hormones, and important mediators in cell signal transduction pathways. Accumulating studies have demonstrated that lipid metabolism disorders are closely related to the pathogenesis of COPD by affecting occurrence and development of the disease (Fahy et al., 2005; Chen et al., 2019; Kotlyarov and Bulgakov, 2021). For example, obesity with high amounts of triglyceride and cholesterol was associated with poorer COPD-related outcomes (lower quality of life, more dyspnea, and more severe COPD exacerbations) (Lambert et al., 2017). In addition, some species of sphingolipids were inversely associated with emphysema, while sphingosine 1-phosphate showed negative association with COPD exacerbation (Bowler et al., 2015). Phospholipids, accounting for forming cell membranes and pulmonary surfactants, were found to be decreased in patients with COPD, which may correlate with pulmonary functions (Lusuardi et al., 1992).

Lipidomics is an “omics” approach, which comprehensively analyzes the full lipid components in various biological samples and can provide significant insights into the understanding of disease pathogenesis. With this technology, fatty acid metabolism was identified to be altered in bronchial epithelial cells of asthmatic patients, leading to an increase in levels of some lipid species [phosphatidylcholine, lysophosphatidylcholine, and bis (monoacylglycero) phosphate] (Ravi et al., 2021). Our previous study found that the levels of lysophosphatidylcholine (LPC) 18:3, lysophosphatidylethanolamine (LPE) 16:1, and phosphatidylinositol (PI) 32:1 significantly dropped in the acute stage compared to the recovery stage in hospitalized patients with COPD exacerbation (Gai et al., 2021). In our study, we aimed to compare the serum lipid metabolite profiles between eosinophilic and non-eosinophilic AECOPD patients, and to explore the association between differential lipid metabolites and patients’ clinical and prognostic features, based on the untargeted liquid chromatography–mass spectrometry (LC-MS) lipidomics technology. We hypothesized that lipid metabolic profiles were different between these two phenotypes of AECOPD, and lipidomics analysis may help elucidate the underlying pathogenesis.

## 2. Materials and methods

### 2.1 Study subjects and data collection

Data were collected from 71 AECOPD patients hospitalized in the Department of Respiratory Medicine and Critical Care Medicine of Peking University Third Hospital from April 2017 to March 2018. These patients met the criteria for the diagnosis of acute exacerbation of COPD, according to the Global Initiative for Chronic Obstructive Lung Disease (GOLD) guidelines (Global Initiative for Chronic Obstructive Lung Disease, 2022). The exclusion criteria were as follows: subjects with other airflow limitation diseases rather than COPD, combination of pneumonia and active pulmonary tuberculosis, severe liver and kidney insufficiency, malignancies, an immunosuppressive condition due to chemotherapy or HIV infection, receiving systemic glucocorticoids due to COPD exacerbation in the past 1 month, and severe trauma or stress reaction.

The clinical data included demographic characteristics, smoking status, comorbidities, presence or absence of hypercapnic respiratory failure [arterial carbon dioxide partial pressure (PaCO_2_) ≥ 50 mmHg], the length of stay (LOS) in the hospital, and requirement for an intensive care unit (ICU) stay or not. The time taken for the next exacerbation was also collected.

A blood routine test and blood biochemical examination were performed in the clinical laboratory of our hospital. The peripheral blood cell count and classification, fibrinogen, D-dimer, total cholesterol, total triglyceride, and other laboratory parameters were recorded. Our patients were grouped according to the percentage of eosinophils (EOS) in peripheral blood. Non-eosinophilic AECOPD was defined as having blood EOS%< 2% and eosinophilic AECOPD as having blood EOS ≥ 2%.

All subjects or their close relatives participating in this study signed an informed consent before data collection. The study procedures were performed in compliance with the Declaration of Helsinki (1964), and the study protocol was approved by the Ethics Committee of Peking University Third Hospital (M2017410).

### 2.2 LC-MS analysis

#### 2.2.1 Serum sample collection and preparation

Fasting (after at least 8 h) peripheral blood samples were collected from patients using vacuum blood collection tubes. The blood samples were left at room temperature for approximately 30 min until complete clotting, and then, the samples were centrifuged at 4°C at 2,500 × g for 15 min. The upper serum samples were extracted and then placed in frozen storage at −80°C.

#### 2.2.2 Lipid metabolite extraction

After being reheated and dissolved, 100 μL of the serum sample was transferred into an EP tube, and then, 480 μL of the extract solution (methyltert-butylether: methanol = 5:1) was added to the sample. After vortexing and mixing for 30 s, the samples were sonicated in an ice water bath for 10 min. After incubating at −40°C for 1 h, the sample was centrifuged at 3,000 rpm for 15 min at 4°C. Then, 350 μL of the supernatant was transferred to a fresh EP tube and vacuum dried. A measure of 200 μL of the solution (DCM: MeOH = 1:1) was added to reconstitute the dried samples. Then, the solution was vortexed for 30 s and sonicated in an ice water bath for 10 min. A measure of 75 μL of the supernatant was placed in a fresh glass vial for LC-MS analysis. A quality control (QC) sample was prepared by mixing an equal aliquot (10 μL) of the supernatants from each subject’s sample.

#### 2.2.3 LC-MS analysis procedure

After lipid metabolite extraction was carried out, an ultra-high-performance liquid chromatograph (ExionLC, AB SCIEX, United States) was used to separate the target compounds using a Phenomenex Kinetex C18 (2.1 mm × 100 mm, 1.7 μm, Phenomenex, United States) liquid chromatography column. High-resolution mass spectrometry data acquisition was performed in information-dependent acquisition (IDA) mode, utilizing a triple TOF 5600 mass spectrometer (AB SCIEX, United States). The data acquisition software application (Analyst TF 1.7, AB Sciex) conducts primary acquisition, followed by automated ion selection and secondary mass spectrometry data collection based on predetermined criteria derived from primary mass spectrometry data. In each cycle, the most intensive 12 precursor ions with intensities over 100 were selected for secondary mass spectrometry scanning. The energy of collision-induced dissociation was 45 eV, and the accumulation time of each secondary spectrum was 50 ms. The ion source parameters are as follows: GS1 60 psi, GS2 60 psi, CUR 30 psi, TEM 600°C, DP 100 V, ISVF 5,000 V (ESI + mode), and −3800 V (ESI − mode).

#### 2.2.4 Data preprocessing and annotation

The mass-to-charge ratio (m/z) and retention time (RT) information of the test samples were determined by liquid chromatography–mass spectrometry (LC-MS), and then, XCMS was used for retention time correction, peak identification, peak extraction, peak integration, and peak alignment. Minfrac was set as 0.5, and the cutoff was set as 0.3. The m/z of the substances in the LipidBlast database was matched with RT. In the qualitative process, the score value of the secondary qualitative metabolites was calculated based on the Euclidean distance and the dot product algorithm, which improved the accuracy of the mass spectrum annotation. XCMS parameters were set as follows: centWave, ppm 10, peak width 5–20, and SN 3; prefiltering step: the metabolites could be retained only if it contained at least three peaks of intensity ≥ 1,000. The function used to calculate the m/z center of the chromatographic peak was wMean, which was the intensity weighted average of the m/z values of the peak. The minimum m/z dimension difference required for peaks with overlapping retention times was −0.001. Then, lipid metabolite identification was achieved through a spectral match using the LipidBlast library. Finally, a total of 2,431 lipid metabolites in the ESI + mode and 1,821 lipid metabolites in the ESI − mode were detected for further multivariate analysis.

### 2.3 Statistical analysis

The baseline data were compared between the patients with the percentage of peripheral eosinophils ≥ 2% and <2% using Student’s *t*-test for continuous and Pearson’s chi-squared test or Fisher’s exact test for categorical variables. The numerical variables were presented as the mean value and standard deviation (SD), while the categorical variables were expressed as numbers and percentages.

Significance of the difference between the two groups was analyzed for each lipid using Student’s *t*-test, and an FDR-adjusted *p*-value < 0.05 was considered significant. The fold changes of each lipid were calculated on the basis of the average in each group. Orthogonal projections to latent structure discriminant analysis (OPLS-DA) was applied to obtain a high level of group separation and a good understanding of the variables responsible for classification, and the first principal component of the variable importance projection (VIP) was obtained. VIP values exceeding 1.0 with an adjusted *p*-value < 0.05 in the Student’s *t*-test were selected to correspond to potential lipid biomarker candidates. To control the possible influences of some factors, such as age, sex, BMI, smoking status, serum total cholesterol, and triglyceride, linear regression was performed for each lipid. The lipids with an FDR-adjusted *p*-value < 0.05 from the Wald statistic were retained as potential lipid biomarkers.

We also checked the associations between the percentage of peripheral eosinophils and other clinical features. Linear regression or generalized linear regression was performed for each potential lipid biomarker and clinical features significantly associated with the percentage of peripheral eosinophils to determine more characteristic lipid metabolites. Then, the least absolute shrinkage and selection operator (LASSO) was applied to downsize these lipid metabolites for discriminating eosinophilic and non-eosinophilic AECOPD, while the largest value of lambda, whose corresponding misclassification error was within one standard error of the minimum misclassification error, known as “1-se” lambda, was defined as the optimal value. A logistic regression model was fitted using these selected markers as the covariates to obtain a combined screening score. The predictability of the model was evaluated by using the area under receiver operation characteristic curve (ROC).

All the analyses were conducted using R version 4.0.5, with the following packages being used: “ropls,” “glmnet,” and “pROC”. A two-sided *p*-value of < 0.05 was considered statistically significant. All data were analyzed anonymously.

## 3 Results

### 3.1 Clinical characteristics

As shown in Table 1, the mean age of our patients was 74.1 ± 9.6 years, and 87.3% of these patients were male, where 59.2% and 31.0% were former and current smokers, respectively. The age, sex proportion, body mass index, tobacco exposure, and comorbidities were not different between eosinophilic and non-eosinophilic AECOPD patients. Serum triglyceride and cholesterol levels were similar between the groups. Eosinophilic AECOPD patients had less hypercapnic respiratory failure, less ICU admission, and shorter LOS in the hospital, as well as a lower fibrinogen level. The time taken for the next exacerbation was not statistically different.

**TABLE 1:** Demographic and clinical characteristics of the study subjects.

Characteristic	Total (*n* = 71)	Non-eosinophilic (*n* = 48)	Eosinophilic (*n* = 23)	*p*-value^a^
Age (years)	74.1 (9.6)	73.7 (10.3)	75.0 (8.0)	0.566
Male	62 (87.3%)	42 (87.5%)	20 (87.0%)	1.000
BMI (kg/m^2^)	22.0 (5.2)	22.0 (5.8)	22.1 (3.7)	0.910
Smoking status				
Never	7 (9.9%)	5 (10.4%)	2 (8.7%)	0.303
Former	42 (59.2%)	31 (64.6%)	11 (47.8%)	
Current	22 (31.0%)	12 (25.0%)	10 (43.5%)	
Smoking index (packs per year)	39.2 (29.7)	37.7 (28.1)	42.2 (33.3)	0.578
Comorbidities				
Hypertension	37 (52.1%)	27 (56.3%)	10 (43.5%)	0.451
Coronary heart disease	11 (15.5%)	8 (16.7%)	3 (13.0%)	1.000
Heart failure	4 (5.6%)	3 (6.3%)	1 (4.3%)	1.000
Diabetes	11 (15.5%)	8 (16.7%)	3 (13.0%)	1.000
Hyperlipidemia	8 (11.3%)	5 (10.4%)	3 (13.0%)	0.708
Eosinophil count (cells/μL)	115 (176)	33 (44)	287 (220)	<0.001
Eosinophil %	1.65 (2.23)	0.43 (0.50)	4.19 (2.29)	<0.001
D-dimer (μg/mL)	0.26 (0.19)	0.28 (0.21)	0.21 (0.13)	0.106
Fibrinogen (g/L)	3.97 (1.29)	4.20 (1.41)	3.48 (0.82)	0.008
Total cholesterol (mmol/L)	3.98 (0.85)	3.95 (0.89)	4.04 (0.80)	0.668
Triglyceride (mmol/L)	1.02 (0.46)	0.96 (0.40)	1.14 (0.55)	0.156
Hypercapinic respiratory failure	24 (36.9%)	22 (45.8%)	2 (8.7%)	0.004
Need for ICU admission	14 (19.7%)	13 (27.1%)	1 (4.3%)	0.027
Time to next exacerbation (months)	27.1 (13.6)	27.1 (13.6)	27.1 (13.9)	0.994

Data are presented as mean (SD) or n (%).

### 3.2 Lipid metabolite profiling difference between eosinophilic and non-eosinophilic AECOPD

**FIGURE 1:**
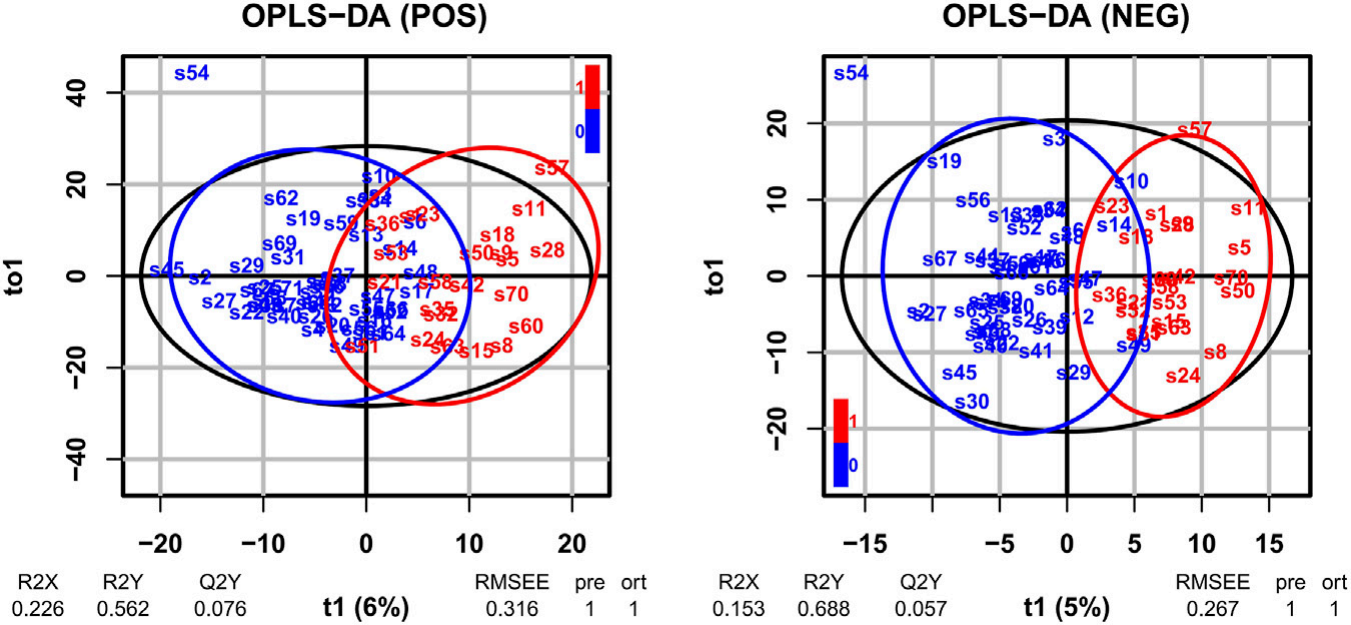
OPLS-DA of LC-MS metabolite profiles between eosinophilic AECOPD and non-eosinophilic AECOPD subjects in electrospray positive ion (ESI+) mode and electrospray negative ion (ESI−) mode, respectively. Red points represented eosinophilic AECOPD subjects, while blue points represented non-eosinophilic AECOPD subjects. OPLS-DA, orthogonal projections to latent structures-discriminate analysis; LC-MS, liquid chromatography-mass spectrometry; AECOPD, acute exacerbation of chronic obstructive pulmonary disease; POS, electrospray positive ion mode; NEG, electrospray negative ion mode.

Log2 transformation was performed before statistics analysis. First, OPLS-DA was performed. As shown in Figure 1, OPLS-DA plots both demonstrated a barely clear separation between eosinophilic and non-eosinophilic AECOPD, with most samples being within the 95% confidential interval with the exception of one non-eosinophilic sample. The R2Y values of the OPLS-DA model in ESI + and ESI − modes were 0.562 and 0.688, respectively, and the Q2Y values were 0.076 and 0.057, respectively. A total of 838 and 616 lipids had a VIP score > 1 in ESI + and ESI − modes, respectively. Subsequently, a *t*-test with FDR-adjusting values and fold changes was conducted to compare the difference between the mean concentrations of lipid species between the two groups. In this process, a total of 32 lipid metabolites (26 in ESI + and 6 in ESI − modes) met both FDR-adjusted *p* < 0.05 and VIP > 1, and were selected as potential candidate metabolites.

**FIGURE 2:**
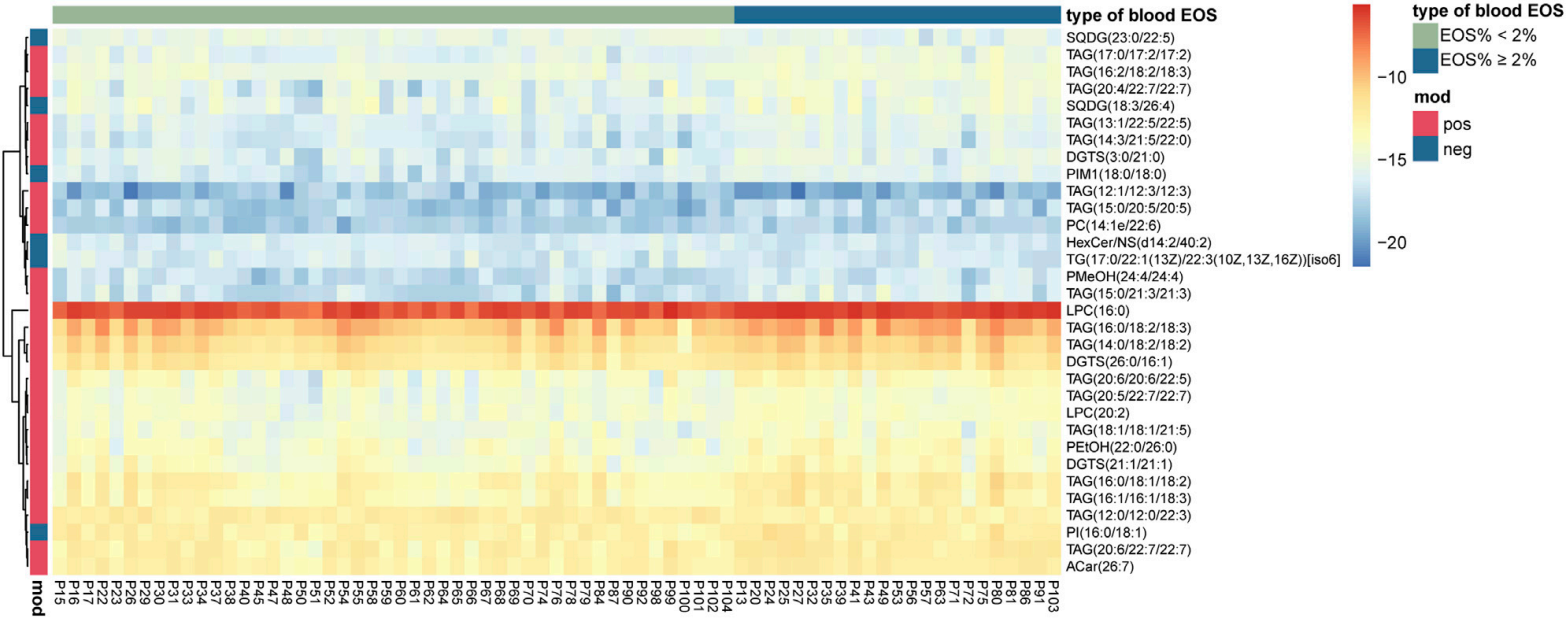
Heat map presenting 32 differential lipid metabolites between eosinophilic AECOPD and non-eosinophilic AECOPD subjects.

To further control the possible influencing factors, such as age, sex, BMI, smoking status, serum total cholesterol, and triglyceride, a linear regression model was structured for each candidate, and lipids with an FDR-adjusted *p*-value < 0.05 from the Wald statistic were retained in this model. It turned out that all the 32 candidate metabolites were retained. Therefore, a total of 32 lipid metabolites (Table 2) were selected as potential biomarkers, and they were mapped to show the clear difference between the two groups (Figure 2).

**TABLE 2:** Differentially expressed metabolites between eosinophilic and non-eosinophilic patients.

Metabolite	Median m/z	Median RT (s)	MS2. score	Mean non-eosinophilic	Mean eosinophilic	log2fc	p.*t*-test	p.t.adj	VIP
Positive-ion mode (ESI +)									
TAG (12:0/12:0/22:3)	795.611	424.092	0.876	2.24 × 10^−4^	1.69 × 10^−4^	−0.405	5.60 × 10^−4^	0.040	2.147
TAG (12:1/12:3/12:3)	647.423	155.660	0.876	3.27 × 10^−6^	1.93 × 10^−6^	−0.756	6.50 × 10^−4^	0.042	2.088
TAG (13:1/22:5/22:5)	933.671	660.639	0.880	1.54 × 10^−5^	2.30 × 10^−5^	0.576	1.23 × 10^−4^	0.022	2.469
TAG (14:0/18:2/18:2)	849.695	655.631	0.976	4.48 × 10^−4^	6.61 × 10^−4^	0.559	6.20 × 10^−4^	0.042	2.258
TAG (14:3/21:5/22:0)	934.675	659.682	0.975	1.26 × 10^−5^	1.93 × 10^−5^	0.615	2.59 × 10^−4^	0.033	2.167
TAG (15:0/20:5/20:5)	907.657	656.299	0.882	5.00 × 10^−6^	8.04 × 10^−6^	0.685	6.48 × 10^−4^	0.042	2.101
TAG (15:0/21:3/21:3)	938.819	643.019	0.515	9.23 × 10^−6^	1.70 × 10^−5^	0.878	5.78 × 10^−4^	0.040	2.092
TAG (16:0/18:1/18:2)	874.699	643.315	0.958	1.50 × 10^−4^	2.48 × 10^−4^	0.723	4.96 × 10^−4^	0.038	2.452
TAG (16:0/18:2/18:3)	870.756	660.559	0.834	9.80 × 10^−4^	1.65 × 10^−3^	0.752	5.56 × 10^−4^	0.040	2.148
TAG (16:1/16:1/18:3)	847.680	637.878	0.903	1.17 × 10^−4^	2.03 × 10^−4^	0.794	3.54 × 10^−4^	0.037	2.341
TAG (16:2/18:2/18:3)	866.673	655.307	0.577	3.17 × 10^−5^	4.09 × 10^−5^	0.369	6.43 × 10^−4^	0.042	2.306
TAG (17:0/17:2/17:2)	863.676	639.317	0.910	2.04 × 10^−5^	3.32 × 10^−5^	0.703	3.13 × 10^−5^	0.015	2.632
TAG (18:1/18:1/21:5)	941.703	660.805	0.885	4.19 × 10^−5^	6.54 × 10^−5^	0.644	4.18 × 10^−4^	0.037	2.081
TAG (20:4/22:7/22:7)	1,017.690	84.972	0.876	2.21 × 10^−5^	3.64 × 10^−5^	0.725	4.10 × 10^−4^	0.037	2.529
TAG (20:5/22:7/22:7)	1,015.664	83.199	0.876	4.07 × 10^−5^	5.96 × 10^−5^	0.552	8.86 × 10^−5^	0.021	2.903
TAG (20:6/20:6/22:5)	991.674	83.242	0.876	6.68 × 10^−5^	1.20 × 10^−4^	0.849	9.28 × 10^−5^	0.021	2.640
TAG (20:6/22:7/22:7)	1,013.656	83.220	0.876	1.79 × 10^−4^	2.74 × 10^−4^	0.610	3.01 × 10^−5^	0.015	2.962
LPC (16:0)	496.340	83.237	0.982	9.99 × 10^−3^	1.36 × 10^−2^	0.448	3.75 × 10^−5^	0.016	3.021
LPC (20:2)	548.359	112.762	0.852	4.67 × 10^−5^	6.71 × 10^−5^	0.523	9.73 × 10^−5^	0.021	2.740
PC (14:1e/22:6)	762.564	315.218	0.811	4.35 × 10^−6^	5.89 × 10^−6^	0.436	4.74 × 10^−4^	0.038	1.110
DGTS (21:1/21:1)	848.683	637.876	0.641	6.72 × 10^−5^	1.20 × 10^−4^	0.838	1.25 × 10^−4^	0.022	2.380
DGTS (26:0/16:1)	850.699	655.426	0.515	2.63 × 10^−4^	3.83 × 10^−4^	0.542	4.42 × 10^−4^	0.037	2.297
DGTS (3:0/21:0)	600.467	83.235	0.620	1.74 × 10^−5^	2.53 × 10^−5^	0.543	7.55 × 10^−4^	0.044	2.356
ACar (26:7)	526.377	112.711	0.587	1.74 × 10^−4^	2.26 × 10^−4^	0.374	3.84 × 10^−4^	0.037	2.866
PEtOH (22:0/26:0)	918.755	640.810	0.796	6.16 × 10^−5^	1.03 × 10^−4^	0.747	2.14 × 10^−4^	0.029	2.037
PMeOH (24:4/24:4)	888.656	628.171	0.947	7.93 × 10^−6^	1.21 × 10^−5^	0.607	3.80 × 10^−4^	0.037	1.982
Negative-ion mode (ESI -)									
HexCer/NS (d14:2/40:2)	972.837	692.336	0.567	1.35 × 10^−5^	1.06 × 10^−5^	−0.353	5.87 × 10^−3^	0.123	2.412
PI (16:0/18:1)	835.519	273.846	0.815	1.81 × 10^−4^	2.48 × 10^−4^	0.452	2.46 × 10^−3^	0.077	1.965
SQDG (18:3/26:4)	947.607	85.781	0.826	2.70 × 10^−5^	4.12 × 10^−5^	0.611	9.92 × 10^−3^	0.156	1.799
SQDG (23:0/22:5)	965.616	449.079	0.876	2.63 × 10^−5^	2.19 × 10^−5^	−0.264	5.88 × 10^−2^	0.311	1.559
TAG [17:0/22:1 (13Z)/22:3 (10Z,13Z,16Z)] [iso6]	979.846	688.173	0.898	1.43 × 10^−5^	1.08 × 10^−5^	−0.400	2.68 × 10^−2^	0.229	2.192

RT, retention time; log2fc, log2 transformed fold change; p. *t*-test, *p*-value of the *t*-test; p.t.adj, FDR-adjusted *p*-value of the *t*-test; VIP, variable importance projection.

Lipid metabolites: TAG, triacylglycerol; LPC, lysophosphatidylcholine; PC, phosphatidylcholine; DGTS, diacylglyceryl trimethylhomoserine; ACar, acylcarnitine; PEtOH, phosphatidylethanol; PMeOH, phosphatidylmethanol; HexCer/NS, hexosylceramide non-hydroxyfatty acid-sphingosine; PI, phosphatidylinositol; SQDG, sulfoquinovosyl diacylglycerol.

In the ESI + mode, the levels of most of triacylglycerols (TAGs), lysophophatidylcholines (LPCs), and diacylglyceryl trimethylhomoserine (DGTSs) were significantly higher in the eosinophilic AECOPD patients than those in the non-eosinophilic patients, except TAG (12:0/12:0/22:3) and TAG (12:1/12:3/12:3). In the ESI − mode, the levels of PI (16:0/18:1), PIM1 (18:0/18:0), and SQDG (18:3/26:4) were significantly higher in the eosinophilic AECOPD patients.

### 3.3 Integration analysis between clinical characteristics and differential lipid metabolites

The correlation analysis between the 32 lipid metabolites and the clinical characteristics of all the AECOPD patients is shown in Supplementary Figure 1. The most significant clinical characteristics correlated with lipid metabolites were body mass index, D-dimer, fibrinogen, total cholesterol, hypercapnic respiratory failure, and ICU admission. We further analyzed the associations between each of these 32 metabolites and the clinical features which were different between the eosinophilic and non-eosinophilic AECOPD, including hypercapnia respiratory failure, need for ICU admission, and fibrinogen. There were nine differential lipid metabolites associated with each of the three features (Table 3). Most of them have a negative association with these three features.

**TABLE 3:** Nine lipid metabolites and their association with clinical features^a^.

Metabolite	Preliminary screening			Eosinophil		Hypercapnic respiratory failure		Need for ICU		Fibrinogen	
	log2fc	p.t.adj	VIP	β	p.adj	OR (95% CI)	p.adj	OR (95% CI)	p.adj	β	p.adj
TAG (20:6/20:6/22:5)	0.849	0.021	2.640	1.186	0.001	0.377 (0.211, 0.675)	0.011	0.433 (0.236, 0.792)	0.028	−0.426	0.004
TAG (20:6/22:7/22:7)	0.610	0.015	2.962	0.807	0.001	0.230 (0.095, 0.554)	0.011	0.224 (0.088, 0.575)	0.023	−0.618	0.005
TAG (20:5/22:7/22:7)	0.552	0.021	2.903	0.731	0.001	0.228 (0.090, 0.578)	0.012	0.198 (0.071, 0.554)	0.023	−0.694	0.004
LPC (20:2)	0.523	0.021	2.740	0.663	0.001	0.259 (0.098, 0.685)	0.024	0.204 (0.064, 0.649)	0.028	−0.771	0.004
TAG (17:0/17:2/17:2)	0.703	0.015	2.632	0.637	0.003	0.264 (0.099, 0.703)	0.024	0.203 (0.059, 0.698)	0.036	−0.689	0.005
DGTS (3:0/21:0)	0.543	0.044	2.356	0.628	0.012	0.200 (0.075, 0.534)	0.011	0.286 (0.121, 0.672)	0.028	−0.686	0.001
LPC (16:0)	0.448	0.016	3.021	0.563	0.001	0.126 (0.036, 0.449)	0.011	0.103 (0.024, 0.439)	0.023	−0.987	0.004
ACar (26:7)	0.374	0.037	2.866	0.498	0.001	0.174 (0.050, 0.607)	0.024	0.124 (0.028, 0.548)	0.028	−1.162	0.001
TAG (16:2/18:2/18:3)	0.369	0.042	2.306	0.341	0.012	0.175 (0.043, 0.712)	0.028	0.104 (0.019, 0.570)	0.033	−0.836	0.025

a: Association of every one-fold increase of a marker with clinical features. Markers were considered the response variable for EOS, while the predictor variable for the other three clinical features.

Abbreviation: EOS, percentage of peripheral eosinophils; RF, respiratory failure; log2fc, log2 transformed fold change; p.t.adj, FDR-adjusted *p*-value for the *t*-test; VIP, variable importance projection; β, log2 transformed fold change after adjusting for age, sex, BMI, smoking status, serum total cholesterol, and triglyceride; p. adj, FDR-adjusted *p*-value.

Lipid metabolites: TAG, triacylglycerol; LPC, lysophosphatidylcholine; DGTS, diacylglyceryl trimethylhomoserine; ACar, acylcarnitine.

**FIGURE 3:**
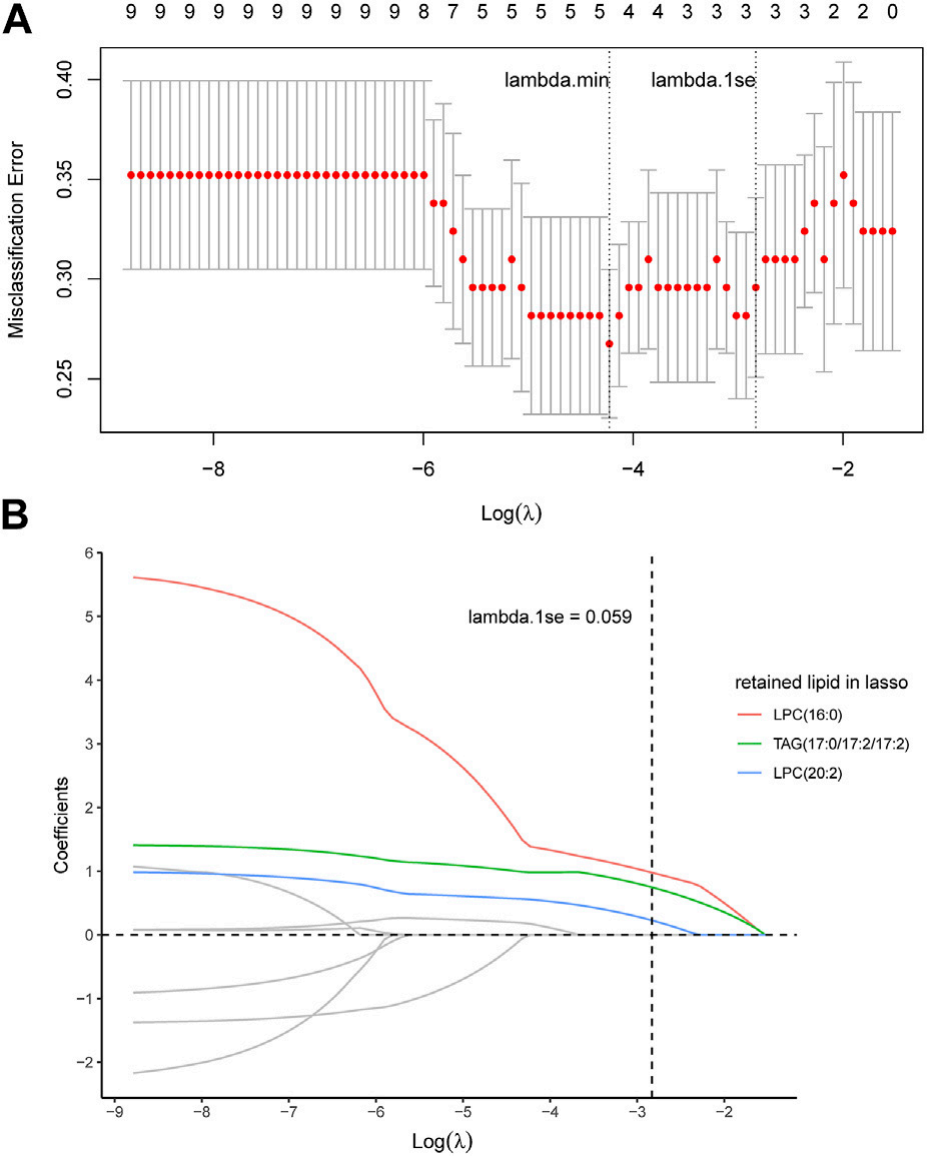
LASSO analysis and cross-validation to identify the most characteristic lipid biomarkers. **(A)** Nine potential lipid biomarkers were included in the model for seven-fold cross-validation, in which lambda.min and lambda.1se were determined. Accordingly, lambda.1se was selected as the optimal value and there were three corresponding lipid metabolites at this optimal value. **(B)** Coefficient profiles of nine potential lipid biomarkers corresponding to different lambda values in LASSO analysis. LPC (16:0), TAG (17:0/17:2/17:2), and LPC (20:2) were retained in this model at lambda.1se = 0.059.

**FIGURE 4:**
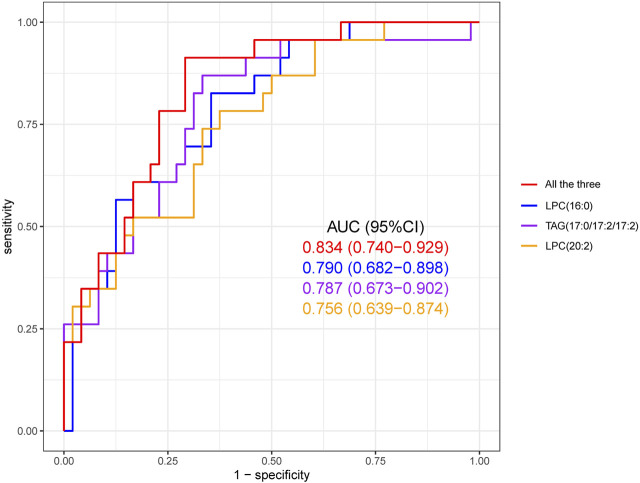
Receiver operating characteristic (ROC) curve of the diagnostic prediction model with three LASSO-selected markers, namely, LPC (16:0), TAG (17:0/17:2/17:2), and LPC (20:2). The combined prediction performance of these three biomarkers was superior to the individual prediction performance of each biomarker.

In order to identify the most characteristic lipid biomarkers for the eosinophilic AECOPD phenotype, LASSO regression was applied to further screen the potential biomarkers. As shown in Figure 3A, all the nine different lipid metabolites associated with the eosinophilic phenotype and clinical features were included in a 7-fold cross-validation (CV) and a CV plot was generated, in which the lambda.min and lambda.1se values were determined. Based on lambda.1se, three lipid metabolites were retained, which were LPC (16:0), TAG (17:0/17:2/17:2), and LPC (20:2) (Figures 3A, B). All of these three lipid metabolites were high in patients with eosinophilic AECOPD. Next, logistic regression models were fitted by individually using each of the three selected markers as the covariate, as well as using them together to obtain the individual screening scores and a combined score, respectively. The predictability of the models was evaluated by the area under ROC curve. As shown in Figure 4, the area under the ROC curve values of LPC (16:0), TAG (17:0/17:2/17:2), and LPC (20:2) were 0.790, 0.787, and 0.756, respectively. In addition, the AUC reached 0.834 (95% CI: 0.740–0.929) when including all the three markers together, which meant this model had an excellent discriminative capacity to distinguish eosinophilic AECOPD and non-eosinophilic AECOPD.

## 4 Discussion

Our study demonstrated the unique characteristics of lipid profiles measured using LC-MS in a group of hospitalized AECOPD patients and indicated that alterations in lipid metabolism were involved in the pathogenesis of the eosinophilic phenotype. In our study, we observed that nine lipid metabolites were significantly associated with the difference in clinical features and prognosis between the eosinophilic and non-eosinophilic phenotypes, which were enriched in triglycerides and phospholipid metabolic pathways and were expected to be potential biomarkers for AECOPD patients and help explain the mechanisms of different clinical outcomes in the eosinophilic phenotype. Furthermore, three lipid metabolites were further screened using LASSO, including LPC (16:0), TAG (17:0/17:2/17:2), and LPC (20:2). Their combination was reliable to predict the clinical prognosis of AECOPD patients in different phenotypes.

Triglycerides are lipids containing three glyceryl groups (Fahy et al., 2005; Liebisch et al., 2020) and are mainly found in the adipose tissue (Chaurasia et al., 2016; Wu et al., 2020). Few studies have explored the characteristics and mechanisms of triglyceride metabolism of eosinophilic COPD. Our data showed that some species of TAGs were higher in those with eosinophilic AECOPD. In previous studies, high triglycerides and low HDL-C played an important role in type 2 inflammation in asthmatic patients (Barochia et al., 2017; Chanachon et al., 2022); the mechanism may be that free fatty acids released by triglycerides promoted inflammation by activating NF-κB signaling in mononuclear cells and enhancing reactive oxygen species generation (Tucker et al., 2020). Additionally, fatty acid synthesis or uptake and subsequent TAG synthesis were also significantly enhanced after inflammatory activation (Tucker et al., 2020). Airway inflammation in eosinophilic COPD and asthma were likely to share a similar mechanism, which could partly explain why some species of TAGs were increased in those with eosinophilic AECOPD. However, the increased levels of these species of TAGs in eosinophilic AECOPD were associated with better clinical outcomes and less systemic inflammation (a lower fibrinogen level) in our study, which seemed to contradict previous studies. A deficiency of lysosomal acid lipase could lead to disruption of triglyceride and cholesterol ester metabolism in alveolar macrophages, leading to respiratory inflammation, tissue remodeling, and emphysema (Lian et al., 2004; Lian et al., 2005). In addition, inflammation also increased the activity of angiopoietin-like protein, an inhibitor of lipoprotein lipase, which further prevented the metabolism of triglyceride-rich lipoproteins and led to elevated triglyceride levels (Lu et al., 2010). Triglycerides were associated with airflow obstruction and wheezing in asthma patients (Fenger et al., 2013; Barochia et al., 2015; Chanachon et al., 2022). However, inconsistent results between previous studies and ours may be related to different study population and disease statuses. In addition, very few studies elucidated the relevant mechanism of the effect of triglyceride species on eosinophil proliferation, activity, and function. The alteration of TAG metabolism and its role in the pathogenesis of eosinophilic COPD need to be further investigated.

Phospholipids are lipids containing phosphoric acid (Fahy et al., 2005; Liebisch et al., 2020) and are the main components of the biological membrane. Phospholipase A2 splits phospholipids into lysophospholipids (a kind of phospholipid containing a single fatty acid) and fatty acids. In our study, LPC (16:0) and LPC (20:2) were increased in eosinophilic AECOPD and associated with fewer hypercapnic respiratory failures, shorter ICU stay, and lower fibrinogen level. The physiological role of LPC in inflammation was complicated. In previous literature, LPCs could exhibit proinflammatory or anti-inflammatory activity under different conditions. Under certain pathophysiological conditions, LPC can be used as a proinflammatory substance. The mechanism may be that LPC-dependent NADPH oxidase can stimulate the production of reactive oxygen species, thus promoting the transformation of pro-cytokines into their mature bioactive forms (such as IL-1β, IL-18, and IL-33), promoting the occurrence of inflammation (Schilling and Eder, 2010). However, under other conditions, some polyunsaturated LPCs (such as LPC 20:4, LPC 20:5, and LPC 22:6) can exhibit anti-inflammatory effects. The mechanism may be to downregulate the formation of pro-inflammatory mediators (such as IL-5, IL-6, NO, 12-hydroxy eicosapentaenoic acid, and LPC16:0-induced PGE2) and upregulate the expression of anti-inflammatory mediators (IL-4 and IL-10) by reducing leukocyte exosmosis and plasma leakage (Riederer et al., 2010; Hung et al., 2011; Hung et al., 2012). In our study, elevated levels of several LPCs were found to have negative correlations with fibrinogen in eosinophilic AECOPD patients, suggesting that systemic inflammatory responses may be weaker and clinical outcomes may be better, and that LPC may have a protective effect in eosinophilic AECOPD patients. Regarding the association between LPC and eosinophilic AECOPD phenotypes, we speculated the mechanism as follows: eosinophils had been demonstrated to express high levels of phospholipase A2 (Blom et al., 1998), which can cleave phosphatidylcholine into LPC and a free fatty acid and increase the level of LPC. Additionally, LPC could induce eosinophils to adhere on and infiltrate into the airway wall (Nishiyama et al., 2004; Zhu et al., 2007). However, most previous studies were based on allergic diseases, such as asthma and allergic rhinitis. The saturated or unsaturated fatty acid chains on LPC species may exhibit different effects on inflammation. Therefore, these issues should be addressed in further studies.

In our study, we also found that eosinophilic AECOPD patients had better clinical outcomes, with shorter hospital stays, fewer cases of respiratory failure, and a lower rate of ICU admission during hospitalization. These results were similar to those found in a meta-analysis. In this analysis, eosinophilic AECOPD (blood eosinophilia ≥ 2% or 0.34 × 10^9^ cells/L) had a better prognosis (lower risk of in-hospital mortality, shorter stay in hospital, and lower risk of arrhythmia) (You and Shi, 2021). Although studies showed a higher risk of readmission (shorter first COPD-related readmissions and an increased number of 12-month COPD-related readmissions) in eosinophilic AECOPD patients, our study did not suggest an increase in the risk of acute exacerbations again (Peng et al., 2021).

This study included the following limitations: 1) since our study results were drawn from a small sample size, further studies are needed to elucidate the relation between lipid metabolism and phenotypes of AECOPD. 2) The Q2Y value in OPLS-DA was relatively low, which meant the estimates from the model were probably on the lower side. This may be due to a possible small difference between the whole lipid profiles of the two groups and the small sample size. However, a combination of several lipids could model well in the discrimination. In future, we would verify the relationship between these lipid metabolites and disease phenotypes in a prospective study. 3) In this study, we did not observe differences in serum lipid metabolic profiles between the two groups before and after the treatment, and it would be of great significance in elucidating the mechanism if we could observe the dynamic changes in lipid metabolites with the treatment time.

In conclusion, our LC-MS analysis demonstrated that patients with eosinophilic AECOPD had a unique serum lipid metabolite profile that could be used to differentiate them from non-eosinophilic AECOPD patients. TAGs and LPCs were significantly increased in eosinophilic phenotypes and associated with less hypercapnic respiratory failure and ICU admission, as well as a lower fibrinogen level, suggesting that these lipid species can serve as biomarkers and play an important role in the pathogenesis of COPD exacerbation. Further studies regarding the mechanisms around lipid metabolism and metabolic pathways will help develop potential therapeutic targets for patients with COPD.

## Data Availability

The raw data supporting the conclusions of this article will be made available by the authors, without undue reservation.
