# Supplementary material for: Differences in the lipid metabolism profile and clinical characteristics between eosinophilic and non-eosinophilic acute exacerbation of chronic obstructive pulmonary disease
Source: Front Mol Biosci. 2023 Jul 12;10:1204985. doi: 10.3389/fmolb.2023.1204985 (PMC10369057; doi:10.3389/fmolb.2023.1204985)
Supplement: Supplementary file 1 [file DataSheet1.PDF]

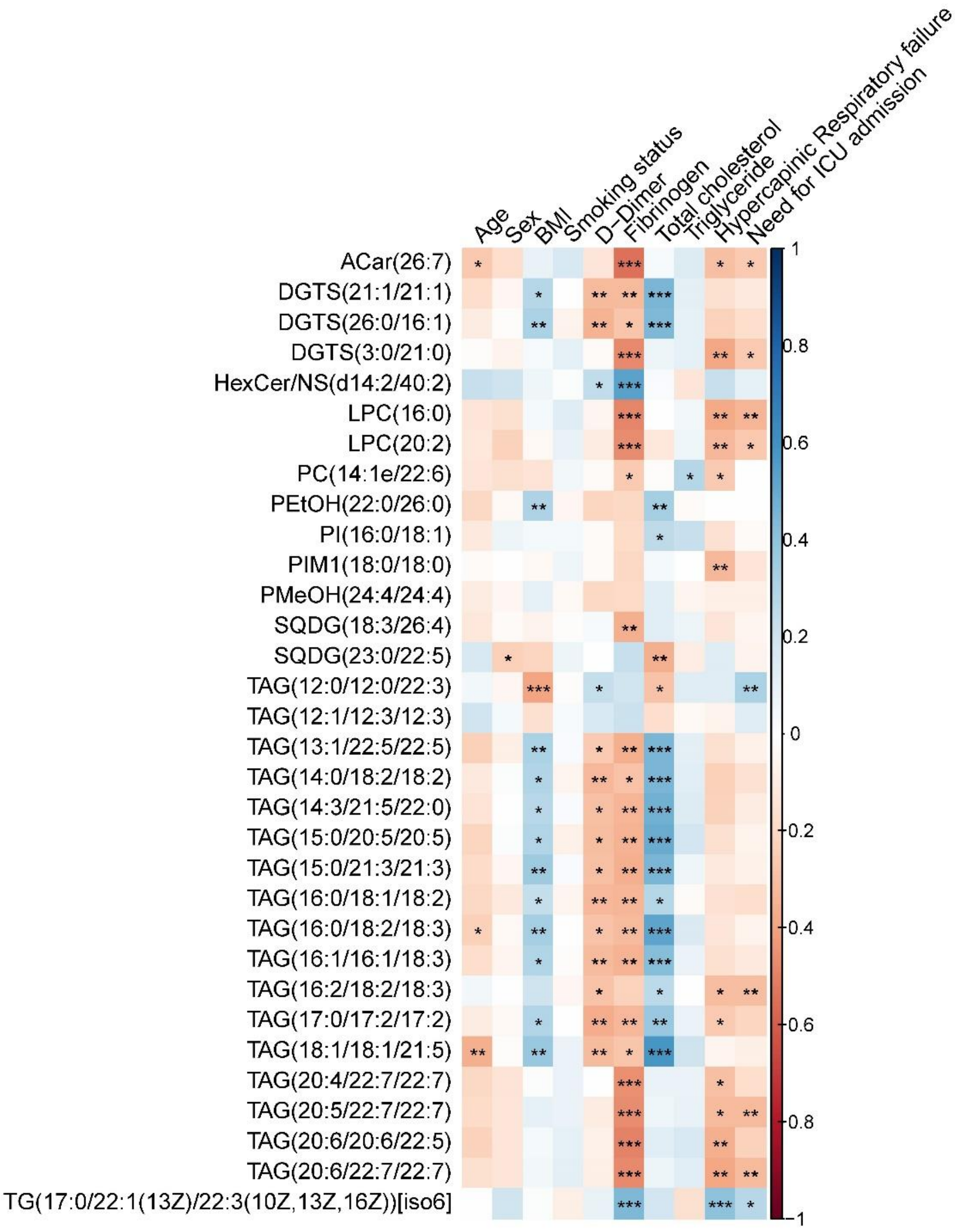

Supplementary Figure 1. Correlation between 32 lipid metabolites and the clinical characteristics of all the AECOPD patients.

*, $P<0.01$; **, $P<0.01$; ***, $P<0.001$.
